# Incidence of postoperative cerebrospinal fluid leaks in endoscopic endonasal transsphenoidal surgery for pituitary adenomas without sellar floor reconstruction: A retrospective observational study from a low-middle-income country

**DOI:** 10.12669/pjms.40.12(PINS).11109

**Published:** 2024-12

**Authors:** Sumira Kiran, Shuja Ikram, Ahtesham Khizar, Muhammad Kaleem Iftikhar, Haseeb Mehmood Qadri, Muhammad Waqas Umer, Khawar Anwar, Asif Bashir

**Affiliations:** 1 Sumira Kiran, MBBS, MS Assistant Professor Neurosurgery, Department of Neurosurgery Unit-I, Punjab Institute of Neurosciences, Lahore, Pakistan; 2 Shuja Ikram, MBBS Postgraduate Resident Neurosurgery, Department of Neurosurgery Unit-I, Punjab Institute of Neurosciences, Lahore, Pakistan; 3 Ahtesham Khizar, MBBS, FCPS Senior Registrar Neurosurgery, Department of Neurosurgery Unit-I, Punjab Institute of Neurosciences, Lahore, Pakistan; 4 Muhammad Kaleem Iftikhar, MBBS Postgraduate Resident Neurosurgery, Department of Neurosurgery Unit-I, Punjab Institute of Neurosciences, Lahore, Pakistan; 5 Haseeb Mehmood Qadri, MBBS Postgraduate Resident Neurosurgery, Department of Neurosurgery Unit-I, Punjab Institute of Neurosciences, Lahore, Pakistan; 6 Muhammad Waqas Umer, MBBS Postgraduate Resident Neurosurgery, Department of Neurosurgery Unit-I, Punjab Institute of Neurosciences, Lahore, Pakistan; 7 Khawar Anwar, MBBS, MS Senior Registrar Neurosurgery, Department of Neurosurgery Unit-I, Punjab Institute of Neurosciences, Lahore, Pakistan; 8 Asif Bashir, MD, FAANS, FACS Professor of Neurosurgery, Department of Neurosurgery Unit-I, Punjab Institute of Neurosciences, Lahore, Pakistan

**Keywords:** Cerebrospinal fluid leak, Developing countries, Endoscopy, Pituitary adenoma, Prolactinoma, Sella turcica

## Abstract

**Objectives:**

To determine the incidence of postoperative cerebrospinal fluid (CSF) leaks after endoscopic endonasal transsphenoidal surgery (EETS) for pituitary adenomas without sellar floor reconstruction (SFR).

**Methods:**

This retrospective observational study was conducted at Department of Neurosurgery, Punjab Institute of Neurosciences (PINS), Lahore, Pakistan from January, 2018 to December, 2022. It is a non-probability based consecutive case series. A total of 316 patients meeting the inclusion criteria were selected.

**Results:**

Among the 316 patients, 102 (32.3%) were male, while 214 (67.7%) were female. Mean age was 40.98 ± 7.8 SD (range 23 to 65 years). Regarding size of pituitary adenomas, 19 (6%) were microadenomas and 297 (94%) were macroadenomas. Overall postoperative CSF (poCSF) leak in our patients was 2.8%. Among cases with poCSF leak, 4 (3.9%) were male and five were female (2.3%) with the P-value of 0.477. Regarding size of pituitary adenomas, only one (5.3%) microadenoma had poCSF leak whereas 8 (2.7%) macroadenomas had poCSF leak with the P-value of 0.432.

**Conclusion:**

Pituitary adenomas can be successfully treated with EETS without SFR in patients who do not experience intraoperative CSF leak.

**List of Abbreviations:**
CSF: Cerebrospinal fluid,
EETS: Endoscopic endonasal transsphenoidal surgery,
ioCSF: Intraoperative CSF,
poCSF: Postoperative CSF,
SFR: Sellar Floor Reconstruction,
T1WI: T1 weighted image,
T1WI C+: T1 weighted image with contrast,
T2WI: T2 weighted image

## INTRODUCTION

Cerebrospinal fluid (CSF) leak is a recognized postoperative complication following transsphenoidal surgery.^1^ The incidence of postoperative CSF (poCSF) leak ranges from 0.5% to 15% in some studies, but most authors report that it ranges from 0.5% to 6%.^2^-^5^ poCSF leak leads to potential morbidities including prolonged hospital stay, headache, meningitis and tension pneumocephalus.^3^ Multiple factors that lead to poCSF leak are poorly defined. poCSF leaks can be minimized in situations of intraoperative CSF (ioCSF) leaks via universal sellar floor reconstruction (SFR). Maintaining the vascularity of the sphenoid mucosal flaps by the use of SFR may make it feasible to reduce postoperative complications such as CSF leak, recurrent sinusitis, meningitis, encephalitis, and pneumocephalus.^6^

There is paucity of research on the incidence of poCSF leaks following endoscopic endonasal transsphenoidal surgery (EETS) for pituitary adenomas without SFR and this served as the basis for our study. In patients without ioCSF leak, EETS without SFR may be the preferred method to achieve less invasiveness, cost-effectiveness, short hospital stays, and fewer complications. Research work from this region of the world is scarce, thus this is a unique study from Pakistan. This study will aid in surgical procedure optimization and assist to avoid overtreatment.

## METHODS

A retrospective observational study was conducted at the Department of Neurosurgery, Punjab Institute of Neurosciences (PINS), Lahore, Pakistan over a period of five years (January, 2018 to December, 2022). This was a non-probability based consecutive case series of 316 patients fulfilling the following criteria:

### Inclusion Criteria:

• Patients clinically and radiologically diagnosed with pituitary adenomas.
• Age ranges from 18 to 65 years.
• Those patients who underwent EETS without SFR.
• Patients with no ioCSF leak.

### Exclusion Criteria:

• Tumor extending into suprasellar region or into third ventricle or parasellar extension.
• Patients who had graft repair for ioCSF leak.
• Revision surgery for residual.
• Lumbar drain placement for CSF leak.

### Data Collection Procedure:

This study included 316 patients with pituitary adenoma who underwent EETS without SFR at the Department of Neurosurgery Unit-III, PINS, Lahore, Pakistan.

In our center, it is a standard procedure to get written informed permission from every participant before utilizing their data for medical research. Their files were reviewed and data was collected in a preformed Google form. Data collected includes the patient’s age, gender, preoperative diagnosis (imaging), size of tumors, surgical approach, intraoperative findings, postoperative diagnosis (histopathology), duration of follow-up and complications especially poCSF leak.

### Ethical Approval:

This study was authorized by our institution’s ethical review board with reference No. 1752/IRB/PINS/Approval/2024, dated March 6, 2024.

### Data Analysis Procedure:

Data was analyzed by using Statistical Package for Social Sciences (SPSS) version 23. For stratification purposes gender, and tumor size were compared with the poCSF leaks and P-values were calculated taking < 0.05 as significant.

## RESULTS

### Age and gender distribution:

**Fig1:**
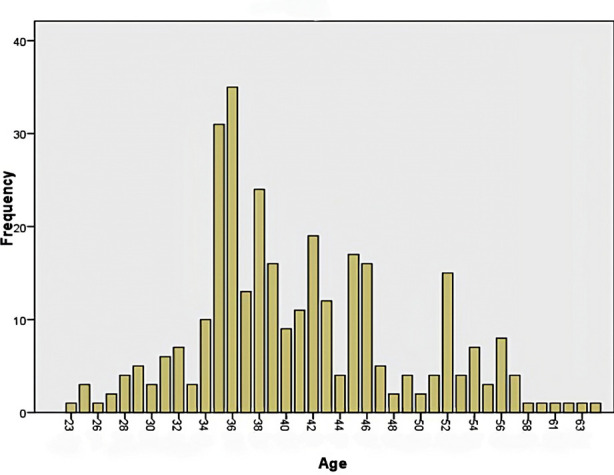
Age distribution among patients (Mean age: 40.98 ± 7.8 SD).

A total of 316 patients meeting the inclusion criteria were chosen for this study. Among these 102 (32.3%) were male and 214 (67.7%) were female as shown in Table-I. Mean age of these patients was 40.98 ± 7.8 SD (range 23 to 65 years, Fig.1).

**Table. I:** Gender distribution among patients

Gender	No. of patients	Percentage (%)
Male	102	32.3
Female	214	67.7
Total	316	100

### Tumor size:

Regarding size of pituitary adenomas, 19 (6%) were microadenomas and 297 (94%) were macroadenomas as given in Table-II.

**Table-II:** Size of pituitary adenomas among patients .

Size	No. of patients	Percentage (%)
Microadenomas	19	6
Macroadenomas	297	94
Total	316	100

### Postoperative CSF leak:

Overall poCSF leak among our patients was 2.8% (9/316) as mentioned in Table-III.

**Table-III:** poCSF leak among patients .

poCSF leak	No. of patients	Percentage (%)
Yes	9	2.8
No	307	97.2
Total	316	100

### Post-stratification results:

Among cases with poCSF leak, 4 (3.9%) were male and five were female (2.3%) with the P-value of 0.477 as shown in Table-IV. Regarding size of pituitary adenomas, only 1 (5.3%) microadenoma had poCSF leak whereas 8 (2.7%) macroadenomas had poCSF leak with the P-value of 0.432 (Table-IV).

**Table-IV:** Post-stratification results.

Parameters		poCSF leak		Total	P-value

		Yes	No		
Gender	Male	4 (3.9%)	98 (96.1%)	102 (32.3%)	0.477
	Female	5 (2.3%)	209 (7.7%)	214 (67.7%)	
	Total	9 (2.8%)	307 (97.2%)	316 (100%)	
Size	Microadenomas	1 (5.3%)	18 (94.7%)	19 (6%)	0.432
	Macroadenomas	8 (2.7%)	289 (97.3%)	297 (94%)	
	Total	9 (2.8%)	307 (97.2%)	316 (100%)	

## DISCUSSION

EETS is now regarded as the first-line therapy for sellar and suprasellar lesions, particularly pituitary adenomas. However, up to 22% of surgeries can result in complications.^7^,^8^ The most frequent EETS complication, a CSF leak, can be found both intraoperatively and postoperatively. PoCSF leaks are less common as a result of improved repair and sellar reconstruction.^7^,^9^ Additionally, there is still uncertainty surrounding the management of poCSF leaks, including the impact of lumbar drains and the timing of reoperation.^7^,^10^ According to earlier research, it was advised to establish a vascularized nasal septal flap before tumor excision or to do SFR since the incidence of CSF leak is higher after the resection of macroadenomas. If there is no arachnoid disruption, CSF leak following EETS of pituitary tumor can be avoided since pituitary adenomas originate in the sellar region, which is outside of the arachnoid membrane and subarachnoid space.^11^ In patients who do not have an ioCSF leak, EETS without SFR may be the preferred approach since it is less invasive, less expensive, results in shorter hospital stays, and has less complications.^6^

There is only one local study, a randomized controlled trial by Majeed MN et al.^6^ Patients were randomized into two groups of 58 each; EETS with SFR was performed in Group A, and EETS without SFR was performed in Group B. There were 52 (89.7%) cases of macroadenoma and 6 (10.3%) cases of microadenoma in each group. On the first postoperative day, CSF leakage was noted in 2 (3.4%) patients of Group A and 2 (3.4%) patients of Group B. As a result, they concluded there was no difference in CSF leakage between the two groups.

Ciric et al.^12^ published data on 958 patients who received EETS by North American neurosurgeons and reported complications of the procedure using a questionnaire in 1997. He further subdivided them as <200, 200-500 and >500 according to the number of cases performed by a surgeon. Incidence of poCSF leak was found to be 4.2%, 2.8% and 1.5% respectively. In contrast to Capabianca et al.^13^, who reported a 2% incidence of CSF leak following EETS in 170 cases, our study found a 2.8% incidence of poCSF leak after EETS.

**Fig.2:**
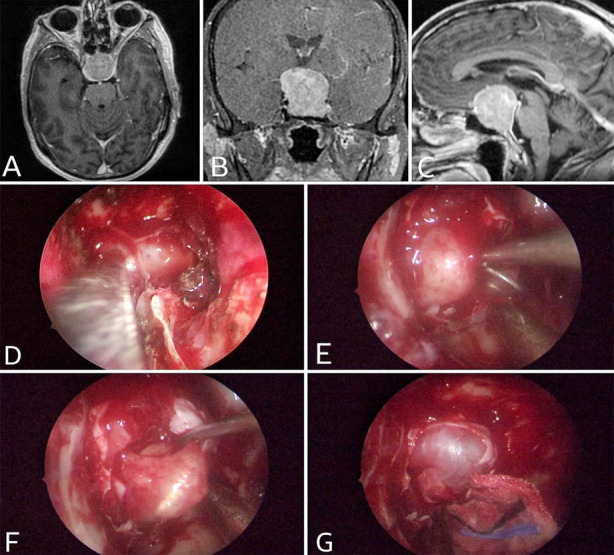
Case. 1: Pituitary macroadenoma, A: Axial T1WI C+, B: Coronal T1WI C+, C: Sagittal T1WI C+, D: Sphenoid exposure, E: Tumor exposure, F: Tumor removal, G: Suprasellar cistern visible.

**Fig.3:**
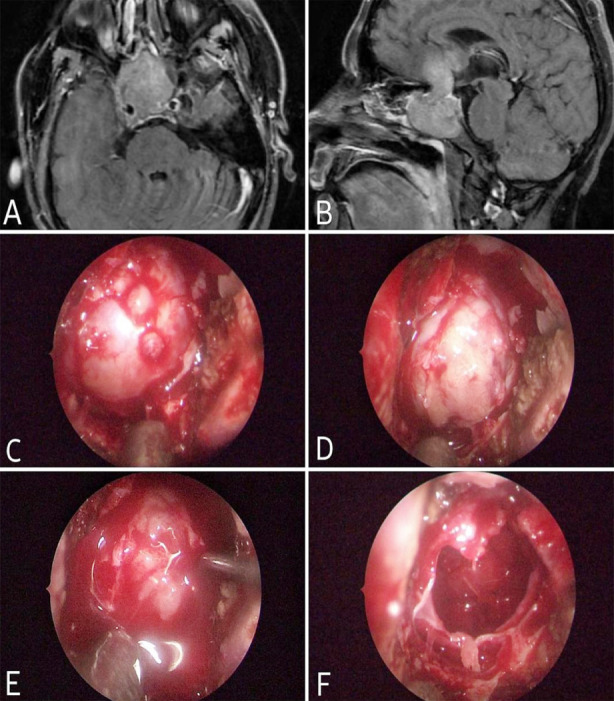
Case. 2: Pituitary macroadenoma, A: Axial T1WI C+, B: Sagittal T1WI C+, C: Post-sphenoidotomy, D: Tumor exposure, E: Tumor removal, F: Tumor cavity after removal.

According to Nishioka et al.^14^, there is a link between age and CSF leak, whereas Romeo et al.^15^ indicates no association between age and CSF leak. However, in the current study, no correlation between age and CSF leak was discovered. Fig.2, 3, and 4 display some of our example cases. None of them had SFR.

**Fig.4:**
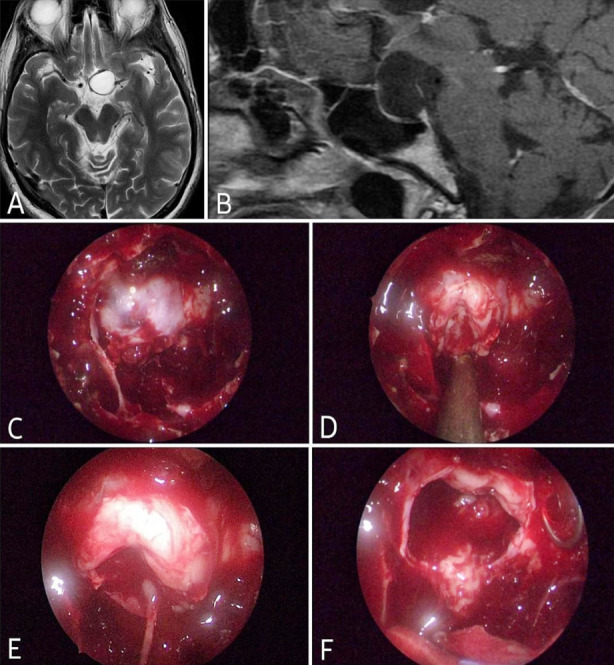
Case. 3: Pituitary apoplexy, A: Axial T2WI, B: Sagittal T1WI, C: Post-sphenoidotomy, D: Tumor exposure, E: Tumor removal, F: Tumor cavity after removal.

We also analyzed the effect of tumor size on poCSF leak. Shiley et al.^1^ observed slightly higher CSF leak after removal of microadenomas versus macroadenomas (6.4% and 4.2%). Perhaps this slightly high incidence of CSF leaks after microadenomas resection in their study was due to the substantial number of ACTH adenomas. Nishioka et al.^14^ had no association between tumor size and CSF leak in his study. In the present study, it appears that the incidence of CSF leak is more in microadenomas versus macroadenomas (5.3% and 2.7%) (Table-IV). This is because of the inequality of the number of cases in our study. Moreover, a slightly lower incidence of poCSF leak was observed following resection of microadenomas versus macroadenomas noted by Zervas et al.^16^ (1.3% vs 3.8%) and Black et al.^4^ (0.9% vs 4.2%).

### Limitations and future recommendations:

The retrospective methodology and small sample size of our study were primarily responsible for its shortcomings. The information collected about patients during hospitalization and follow-up was primarily derived from their medical records. As a result, bias was unavoidable when analyses were carried out using partial and inconsistent data. Future research should employ longitudinal studies to examine the long-term effects and variations in the course of poCSF leak following pituitary adenomas treated with EETS without SFR in patients who did not experience ioCSF leak.

## CONCLUSION

When there is no ioCSF leak, the results of incidence of poCSF leak after EETS for pituitary adenomas without SFR are not significantly different. This emphasizes the importance of EETS without SFR and safeguarding the patients from overtreatment.

### Authors’ Contribution:

**SK:** Conception and Design of study, Data acquisition.

**SI**: Manuscript drafting and Literature review.

**AK:** Data analysis, Interpretation of data, Literature review and Critical review of manuscript.

**MKI, HMQ, MWU, and KA:** Data collection, Literature review and Manuscript drafting.

**AB:** Supervision and Critical Review.

All the authors have read and approved the final manuscript and are responsible and accountable for the accuracy and integrity of the work.
